# Simulating the effect of flowering time on maize individual leaf area in contrasting environmental scenarios

**DOI:** 10.1093/jxb/eraa278

**Published:** 2020-06-11

**Authors:** Sebastien Lacube, Loïc Manceau, Claude Welcker, Emilie J Millet, Brigitte Gouesnard, Carine Palaffre, Jean-Marcel Ribaut, Graeme Hammer, Boris Parent, François Tardieu

**Affiliations:** 1 Univ. Montpellier, INRAE, Montpellier, France; 2 Biometris, WUR, Wageningen, The Netherlands; 3 Univ. Montpellier, INRAE, CIRAD, Institut Agro, UMR AGAP, Montpellier, France; 4 INRAE, UE 0394, SMH Maïs, Centre de recherche de Bordeaux Aquitaine, Saint-Martin-De-Hinx, France; 5 Integrative Breeding Platform, CGIAR, Texcoco, Mexico; 6 The University of Queensland, Queensland Alliance for Agriculture and Food Innovation, Centre for Crop Science, Brisbane, QLD, Australia; 7 CSIRO Agriculture and Food, Australia

**Keywords:** Drought, genetic variability, leaf growth, light, model, temperature, whole plant

## Abstract

The quality of yield prediction is linked to that of leaf area. We first analysed the consequences of flowering time and environmental conditions on the area of individual leaves in 127 genotypes presenting contrasting flowering times in fields of Europe, Mexico, and Kenya. Flowering time was the strongest determinant of leaf area. Combined with a detailed field experiment, this experiment showed a large effect of flowering time on the final leaf number and on the distribution of leaf growth rate and growth duration along leaf ranks, in terms of both length and width. Equations with a limited number of genetic parameters predicted the beginning, end, and maximum growth rate (length and width) for each leaf rank. The genotype-specific environmental effects were analysed with datasets in phenotyping platforms that assessed the effects (i) of the amount of intercepted light on leaf width, and (ii) of temperature, evaporative demand, and soil water potential on leaf elongation rate. The resulting model was successfully tested for 31 hybrids in 15 European and Mexican fields. It potentially allows prediction of the vertical distribution of leaf area of a large number of genotypes in contrasting field conditions, based on phenomics and on sensor networks.

## Teaser

Flowering time affects all processes governing whole-plant leaf area, namely rates, durations, and sensitivity to environmental conditions. Genotype dependency was assessed via large phenomic datasets in field and controlled conditions.

## Introduction

Optimal use of genetic resources is required for food security in a changing climate (Tester and Langridge, 2010; IPCC, 2014). Genomic prediction can assess the performances of thousands of new varieties based on genotypic information, but faces the difficulty of large variations in yield depending on local environmental conditions (Technow *et al.*, 2015; Millet *et al.*, 2016). The rapid development of sensor networks and of environmental grids makes it possible to characterize environmental conditions in any field (Chenu *et al.*, 2013; Harrison *et al.*, 2014). This information can be combined with the genomic prediction of the sensitivity of individual genotypes to environmental conditions, thereby making possible the prediction of the yield of hundreds of genotypes in hundreds of fields (Millet *et al.*, 2019). However, poor prediction of leaf area is often a cause of inaccurate simulations, as shown by comparison of 27 crop models (Martre *et al.*, 2015). This is particularly true if the aim of modelling is to predict the genotype×environment interaction in a range of climatic conditions, rather than to predict the yield of a reference genotype in this range (Parent and Tardieu, 2014). Indeed, current crop models do not handle all the richness of phenomic information that characterizes individual genotypes (Parent and Tardieu, 2014; Wu *et al.*, 2019), in particular the genetic variability of leaf development, of leaf expansion, and of their sensitivity to environmental cues. We argued that the modelling of the growth dynamics of individual leaves is necessary if one wants to take into account the genetic variability of individual processes, which is difficult to do in a ‘big leaf’ model (Hammer *et al.*, 2010; Parent and Tardieu, 2014).

Considerable genetic variability exists for the traits governing leaf area and plant architecture. First, the final leaf number (*N*_final_) is closely related to the duration (number of plastochrons) from plant emergence to the floral transition of the shoot apex, but also to flowering time (Parent *et al.*, 2018). Hence, flowering time indirectly affects final leaf area, the vertical distribution of leaf area, and that of light interception (Perez *et al.*, 2019). Most models take these effects into account via the duration of leaf growth as a whole. The model of Lizaso *et al.* (2003) uses the leaf rank and the area of the largest leaf as input variables, so it indirectly takes into account the *N*_final_ and the duration between germination and flowering times. However, a more generic approach is needed to take into account the genetic control of leaf number. A second trait affecting leaf area and plant architecture is leaf elongation rate (LER) and its distribution along leaf ranks. It varies greatly between maize genotypes in panels of diversity or in populations of recombinant inbred lines, with strong genetic controls of both the maximum LER and response to water deficit (Welcker *et al.*, 2011; Dignat *et al.*, 2013). The third trait driving leaf area is leaf width and its distribution, which also has a large genetic variability controlled by genome loci and environmental conditions that are essentially independent of those controlling leaf elongation (Lacube *et al.*, 2017). In Chenu *et al.* (2008), the effects of quantitative trait loci (QTLs) of responses of leaf elongation to soil water deficit were inserted into a model of leaf growth, resulting in simulations of leaf area in different climatic conditions (Chenu *et al.*, 2009). However, this study was a proof-of-concept study rather than an attempt to develop a multigenotype/multienvironment model because it did not consider the genetic and environmental variabilities either of plant development or of leaf widening. In addition, this model involved a high number of genotypic parameters, some of which were conceptual rather than measureable in a phenotyping platform (e.g. the date of the end of juvenile phase).

We aimed here to develop a model able to simulate the genetic variability of whole-plant leaf area in contrasting environmental conditions, taking into account the genetic variability of underlying traits in a large range of maize genetic material, namely flowering time, the distribution of leaf elongation and widening, and the responses of these traits to environmental conditions such as temperature, light, evaporative demand, and soil water status. For that, we investigated the rules governing the diversity of the distributions of leaf length and width along leaf ranks and translated our findings into model equations. Photoperiodic effects were not analysed here because the temperate genetic material is mostly photoperiod insensitive. A diversity panel of 127 lines with a large variability of flowering time was used to analyse, in five fields, the relationship between leaf rank and final leaf length and width (Table 1, Dataset B). A detailed study of two hybrids with contrasting leaf number (Dataset A) was analysed in one field for identification of the beginning and end of leaf elongation and widening at each leaf rank. Then we used a panel of 14 hybrids with a restricted window of flowering time to derive model parametrization, in two experiments in the field and in a phenotyping platform (Datasets C and D). Next, the model was tested for one of the 14 hybrids in 14 fields and for the 14 hybrids in one field (Datasets E and F). Finally, we tested the model for a limited number of lines with later flowering time in a field in Mexico. This resulted in a flexible model that applies to a large range of genotypes with contrasted earliness. This model was developed as an independent executable component in the BioMA software framework (http://www.biomamodelling.org), interoperable in different crop models, and is freely available to the whole community (Manceau *et al.*, 2020). This potentially allows one to compare the advantages of traits or associated alleles carried by real or virtual genotypes in a range of environmental conditions, and to define ideotypes for specific sets of environmental conditions (Rötter *et al.*, 2015; Gouache *et al.*, 2017; Parent *et al.*, 2018; Tardieu *et al.*, 2018).

**Table 1.** Datasets used in this study

	Dataset	Location		Years	Genotypes	Measured variable	Frequency	Repetition	Figures
Model development	A	Field	Mauguio, France	1998	2 hybrids (Déa/Volga)	Leaf length and width Phenology	16 dates 16 dates	10 plants per date	Figs 3, 4; Supplementary Figs S2,S4
	B	Field	Network (5 sites): France (3 sites), Mexico, and Kenya	2006–2007	127 maize lines	Leaf length and width Phenology	3 dates pre- flowering	3 plants per line	Figs 1, 2, 5; Supplementary Fig. S3
Model parameterization	C	Greenhouse	Montpellier, France	2016	Subset of the DROPS panel (14 hybrids)	Leaf length and width Phenology	Every second day (20 dates)	3 plants per hybrid	Supplementary Fig. S1
	D	Field	Saint-Martin de Hinx, France	2016	Subset of the DROPS panel (14 hybrids)	Leaf length and width Phenology	Flowering time	3 plants per hybrid	–
Model test	E	Field	Network, Europe (14 sites)	2011–2013	Reference hybrid B73×UH007	Leaf length and width Phenology	3 dates pre- flowering	10 plants per site	Supplementary Fig. S7
	F	Field	Mauguio, France	2016	Subset of the DROPS panel (14 hybrids)	Leaf length and width Phenology	3 dates pre- flowering	3 plants per line	Fig. 6
	G	Field	Tlaltizapan Mexico	2006	Subset of the P1P2 mapping population (17 lines)	Leaf length, Phenology	3 dates pre- flowering	3 plants per line	Supplementary Fig. S6

## Materials and methods

### Time course of leaf development and growth in two hybrids (Dataset A)

A field experiment was carried out in Mauguio, France (Table 1, Dataset A) in a field (thousands of plants) sown with the two single cross-hybrids Déa and Volga that differ in flowering time and final leaf number (*N*_final_) (16 and 19, respectively). For each hybrid, we labelled 500 plants which emerged on the same day. Among those, 15 plants were chosen as references and scored every second day for the number of leaves which had appeared and those which were ligulated. Leaves were numbered acropetally from the plant base, and the first true leaf was labelled as leaf 1. Leaves 5 and 10 were labelled once fully expanded to facilitate counting the leaves of higher ranks. Ten plants of the 500 were selected every second day, with phenological stages similar to the mean of the 15 reference plants of the corresponding hybrid. These plants were dissected so all leaves, including those hidden in the whorl, were made visible. The length of all leaves was measured as the distance between the leaf insertion point and the leaf tip. Their width was measured as the maximum width of the considered leaf, observed a few centimetres from the leaf insertion point (Muller *et al.*, 2001). Light was measured every hour with photosynthetic photon flux density (PPFD) sensors; air temperature and relative humidity were measured every fifth minute in ventilated shelters for calculation of air vapour pressure deficit (VPD_air_), and stored every hour. Meristem temperature was measured every hour with fine thermocouples inserted in the apical meristem of 10 plants. The time courses of leaf length and width were calculated as a function of thermal time (°Cd) calculated as in the APSIM model (Hammer *et al.*, 2010) with the smoothing function loess (local polynomial regression fitting), package ‘stats’ in R (R Development Core Team, 2011), parameter ‘span’ set at 0.5. The timings of the beginning and end of leaf elongation were estimated from these smoothed curves as 5% and 95% of final dimensions for each leaf rank (see Supplementary Fig. S4 at *JXB* online). A similar analysis was performed for leaf width.

### Final leaf length and width in a diversity panel of 127 maize lines differing in final leaf number in contrasting environmental conditions (Dataset B)

We analysed 127 maize lines representative of the maize breeding pools (dent, flint, subtropical, and tropical, Supplementary Tables S1, S2) in six field experiments, four in France, one in Mexico, and one in Kenya (Dataset B, Table 1; Supplementary Tables S1, S2). Initially, nearly all lines were present in all sites, but severe problems of maladaptation were obvious in many genotypes that could not be retained in the analysis. Eventually, data from six subtropical and tropical lines were kept in Mexico and Kenya, and data from three subtropical lines were kept in Europe in addition to temperate material (117 lines in total, Supplementary Table S2). N_final_ of these lines ranged from 12 to 25. In each experiment, the experimental design was an alpha lattice with two replicates per treatment, clustered by maturity groups (early, semi-early, and late) to avoid that plots with tall and short plants neighbour each other in the field. Each maturity group was sown at different dates, in such a way that flowering time was simultaneous (within 7 d) for all groups. Plots were 6 m long, with 0.8 m between rows and a plant density of 5–7 plants m^–2^, without raw soil between plots. Light, air temperature, relative humidity (RH), and wind speed were measured as above at <300 m from each experiment, at 2 m height over a reference grass canopy. Soil water potential was measured every day with two series of tensiometers at 30, 60, and 90 cm depths in each field. The final length and width of every second leaf were measured in 10 plants per plot, with the same procedures as above. Genotypes were clustered according to N_final_. Profiles of mean final leaf length and width were calculated for each class of N_final_ by using the smoothing function loess in R (span=0.75).

### Parameters for leaf development and leaf expansion in 14 maize hybrids (Dataset C)

An experiment was carried out in the phenotyping platform PhenoArch (Montpellier, France, https://www6.montpellier.inra.fr/lepse/M3P), presented in detail in Alvarez Prado *et al.* (2018). We considered here 14 maize F_1_ crosses of 14 dent lines with a flint tester, with a restricted range of flowering time, selected for maximizing the genetic diversity among the 254 hybrids of the experiment. Plants were grown in well-watered or water deficit conditions with two replicates per genotype and treatment (Table 1, Dataset C; details in Supplementary Table S3). For each plant, 13 RGB images (2056×2454 pixels) were taken every night (one from the top and 12 side images with a 30° horizontal rotation) and processed as in Cabrera-Bosquet *et al.* (2016). Temperature and VPD were recorded every 15 min in eight sites of the greenhouse. Plants were grown in 9 litre PVC pots filled with a substrate composed of a mixture of clay and organic compost (30/70 volume). In well-watered conditions, soil water content was maintained at retention capacity in each pot by applying the exact amount of water lost by transpiration, keeping the soil water potential at –0.05 MPa (Alvarez Prado *et al.*, 2018). In the water deficit treatment, watering was withdrawn from the appearance of leaf 8 onwards, until soil water potential reached a target soil water potential of –0.4 MPa. This took from 5 d to 8 d depending on individual plants. Soil water potential was then maintained for several days by daily irrigation. A second dry-down period was applied to plants until soil water potential reached –0.6 MPa (Alvarez Prado *et al.*, 2018). The number of leaves in which tips or ligules had appeared were scored every second day for all plants in the experiment. This allowed calculation of the genotypic parameters a_tip_, b_tip_, a_ll1_, and b_ll1_ presented in Box 1, Equations 1–7. Leaf area was estimated by image analysis every second day as in Alvarez Prado *et al.* (2018). The increase in leaf area (m^2^ °Cd^–1^) in well-watered conditions was considered as the maximum genotypic leaf expansion rate at the considered phenological stage. The sensitivity of leaf expansion to soil water deficit (m^2^ °Cd^–1^ MPa^–1^) was calculated as the slope of the regression between the leaf expansion rate and soil water potential, over a period from the appearance of leaf 8 to that of leaf 14, normalized by leaf expansion rate in well-watered conditions in such a way that the *y*-intercept was 1 for all hybrids.

**Box 1. Synthesis of equations and parameters of the model**

**Table:** 

Process	Equation	Parameter	Description	Value
Final leaf number	**Measured**	N_final_	Maximum number of leaves	Genotypic
Tip appearance	***tt***_**tip**_ **: thermal time at the appearance of leaf *n*** Equation 1: *tt*_tip_(*n*)=a_tip_×*n*+b_tip_	a_tip_	Slope of the regression of thermal time with tip appearance (i.e. phyllochron, thermal time between subsequent leaf tip appearances)	Genotypic
		b_tip_	Intercept of the regression of thermal time with tip appearance	Genotypic
Beginning of linear expansion	***tt***_**bl**_ **: thermal time at the beginning of elongation** Equation 2: If *n≤*N_bl___lim_*tt*_bl_(*n*)=*tt*_tip_(*n*) Equation 3: If *n*> N_bl_lim_*tt*_bl_(*n*)=a_bl_×*n*+b_bl_ a_bl_=*k*_bl_ a_tip_ b_bl_=b_tip_+N_bl___lim_×a_tip_×(1–*k*_bl_)	*k*_bl_	Ratio between leaf appearance and linear expansion for the last leaves	0.708
		N_bl___lim_	Transition between first and last leaves for beginning of leaf linear expansion	6
Ligule appearance	***tt***_**ll**_: **thermal time at the appearance of ligule** Equation 4: If: *n≤*α _ll_×N_final_*tt*_ll_(*n*)=a_ll1_×*n*+b_ll1_ Equation 5: If: *n*>α _ll_×N_final_*tt*_ll_(*n*)=a_ll2_×*n*+b_ll2_ a_ll2_=*k*_ll_×a_ll1_ b_112_=b_ll1_+a_ll1_×α _ll_×N_final_×(1–*k*_ll_)	a_ll1_	Slope of the regression of thermal time with ligulation (i.e. thermal time between subsequent leaf ligule appearances)	Genotypic
		b_ll1_	Intercept of the regression of thermal time with ligulation	Genotypic
		*Kbl*_ll_	Ratio between the two ligulation slopes with thermal time	0.454
		α _ll_	Transition between the two linear parts describing leaf ligulation with thermal time relative to N_final_	0.52
End of linear expansion	***tt***_**el**_ **: thermal time at the end of linear elongation** Equation 6: If *n≤*N_final_–N_last_+1 *tt*_el_(*n*)=*tt*_ll_(*n*)–a_lag_×*n* Equation 7: If *n*>N_final_–N_last_+1 *tt*_el_(*n*)=*tt*_el_(*n*–1)	N_last_	Number of last leaves that finish their expansion at the same thermal time	2
		a_lag_	Relative thermal time difference per leaf between ligulation and end of expansion	5.4
Beginning and end of widening	***tt***_**beg,w**_ **and *tt***_**end,w**_ **: thermal time for beginning and end of widening** Equation 8: *tt*_beg,w_ (*n*)=*tt*_bl_ (*n*) Equation 9: *tt*_end,w_ (*n*)=*tt*_el_ (*n*)–lag_w_	lag_w_	Thermal time lag between ends of leaf elongation and widening	39
Leaf elongation	**L: leaf length** Equation 10: $L\left(n,d\right)=\underset{emergence}{\overset{d}{\sum }}\;LER\left(n\right)$ LER: leaf elongation rate **LER**_**norm**_**: normalized maximum leaf elongation rate (normalized by maximum rate of leaf 6)** Equation 11:$LER_{norm}(n)=\theta_{L}*e^{\frac{-{\left(n-B_{L}\right)}^{2}}{2*G_{L}{}^{2}}}\;$$\theta_{L}=\frac{\;1}{\;\;\;e^{\frac{-{\left(6-B_{L}\right)}^{2}}{2*G_{L}{}^{2}}}}\;\;\;$ B_L_=β _L_×N_final_ G_L_=σ _L_×N_final_	a_6_	Maximum leaf elongation rate of leaf 6	Genotypic
		b	Sensitivity of leaf elongation rate to vapour pressure deficit	Genotypic
		c	Sensitivity of leaf elongation rate to soil water deficit	Genotypic
		β _ L_	Coefficient determining the rank of the leaf with maximum growth relative to final leaf number	0.68
	Equation 12: **If *tt***_**bl**_**<*tt*<*tt***_**el**_: LER(*n*)=LER_norm_(*n*)×(a_6_+b VPD+cPSI)× ∆*tt* VPD: vapour pressure deficit (kPa) PSI: soil water potential (MPa) ∆*tt*: equivalent thermal time of day d (°C.d) *tt*: cumulated thermal time at day d (°C.d)	σ _L_	Coefficient determining the skewness of the curve or potential leaf growth relative to final leaf number	0.46
Leaf widening	**W: leaf width** Equation 13: W(*n*,*d*)=W_base_(*n*)+RAD_effect_(*d*) W_base_: leaf width at intercepted light of 0.15 MJ RAD_effect_: effect of intercepted light on leaf width Equation 14: $RAD_{effect}(d)={\text{ r}}_{\text{RAD}}*\left(RAD_{i}(d)-{\text{RAD}}_{\text{base}}\right)$ RAD_i_: mean daily plant intercepted radiation from *tt*_beg,w_ to *tt* Equation 15: $W_{\;base}(n)=W_{6}*e^{\frac{-{\left(\;\;\;n\;\;\;-\;\;\;B_{W}\right)}^{2}}{2*{G_{W}}^{2}}}$ B_W_= β _ W_×N_final_ G_W_=σ _W_×N_final_	RAD_base_	Base value for radiation effect on leaf widening	0.15
		W_6_	Base leaf width of leaf 6	Genotypic
		*r*_RAD_	Sensitivity of leaf widening to intercepted radiation	Genotypic
		β _W_	Coefficient determining the rank of the leaf with maximum base width relative to final leaf number	0.41
		σ _W_	Coefficient determining the skewness of the curve of base width relative to final leaf number	0.69

Equations are presented with their respective numbers in the text, in which variable names are presented. Parameter names are the same as in the text. Parameter values are provided here when they are not genotype specific (common to all studied genotypes, white cells). They are indicated as ‘genotypic’ when they need to be measured for each genotype (grey cells).

Our model uses parameters of elongation of leaf 6, which can be measured with high precision (Sadok *et al.*, 2007), rather than parameters at the whole-plant scale that are easier to score at high throughput. We tested the correspondence between both methods, with an experiment performed in the phenotyping platform Phenodyn [Montpellier, France (Sadok *et al.*, 2007) https://www6.montpellier.inra.fr/lepse/M3P] on four of the 14 hybrids. Three plants of each hybrid were grown under either well-watered conditions or progressive water deficit. The LER of leaf 6, environmental conditions, and soil water content were measured every 15 min. Soil water potential was calculated from soil water content via a water release curve. The maximum LER of leaf 6 was estimated for each genotype as the mean LER of well-watered plants during the night (from 20.00 h to 06.00 h). The sensitivity of the LER to soil water potential was estimated for each hybrid by fitting a linear regression between the mean LER from 02.00 h to 06.00 h and the mean soil water potential sensed by plants for the same period of time. The two methods provided well-related sensitivities (Supplementary Fig. S1), so we used sensitivities obtained in the PhenoArch platform, which is less labour intensive, in the rest of the study after scaling values. Because the sensitivities of the LER to evaporative demand and soil water deficit are highly correlated (Parent *et al.*, 2010), we used the relationship found in Welcker *et al.* (2011) to calculate values of the genotypic parameter b (Box 1) from that of parameter c obtained as above (relationship b=0.69–2.3c, *R*^2^=0.44).

### Final leaf number in the same 14 maize hybrids (Dataset D)

The N_final_ was measured in a field experiment in Saint Martin de Hinx (France) for four plants per hybrid (Dataset D, Table 1). Plants were grown in well-watered conditions at a density of 10 plants m^–2^. Leaf number was measured as above; its genotypic mean was calculated based on four plants. Details on environmental conditions are given in Supplementary Table S3.

### Parameters for leaf widening in the same 14 maize hybrids (Datasets C and D)

Parameter values of leaf widening and sensitivity to intercepted radiation for the same 14 hybrids were calculated from Datasets C and D (Table 1; Supplementary Table S3 for environmental conditions). In both datasets, the final width of leaf 6 was measured as in Lacube *et al.* (2017). The sensitivity to intercepted radiation (genotype-specific parameter r_rad_, Box 1) was calculated by fitting a linear regression on final width versus intercepted radiation during the period of time when the corresponding leaf was widening. We used this sensitivity to normalize the width of leaf 6 in all experiments. A genotype-specific width of leaf 6 was calculated for a reference intercepted radiation at a standard light of 1.5 MJ m^–2^ (parameter W_6_, Box 1), thereby removing the effect of intercepted radiation via Equation 12 (Box 1).

### Model test (Datasets E and F)

A test of the model was performed based on two field datasets. In Dataset E (Table 1), the final leaf lengths and widths of all leaves of the reference hybrid B73×UH007 were measured in both water deficit and well-watered conditions in 14 experiments presented in Millet *et al.* (2019), in eight field sites from 2011 to 2013 spread along a climatic transect in Europe (Supplementary Table S3). In Dataset F (Table 1), the 14 parameterized hybrids were analysed in Mauguio in 2016, with the same measurements (hybrid names in Supplementary Table S4). In both cases, light, air temperature, RH, rainfall, and wind speed were measured every hour in each experiment. Light was measured with PPFD sensors or pyranometers depending on local practices; air temperature and RH were measured in ventilated shelters. Soil water potential was measured from every second hour to every fourth day at 30, 60, and 90 cm depths in watered and rainfed microplots sown with the reference hybrid B73×UH007 with three and two replicates, respectively. Meristem temperature (*T*_meristem_) was calculated every hour in each field, based on a simplified energy balance (Millet *et al.*, 2016). The amount of light intercepted by the reference hybrid during a phenological phase was obtained in each experiment by simulations using the crop model APSIM with climatic data for the experiment as in Lacube *et al.* (2017).

Seventeen recombinant inbred lines of the population P1P2 resulting from a cross between two tropical lines with contrasting sensitivities to water deficit (Welcker *et al.*, 2007) were scored by CIMMYT for leaf length along the stem in a field in Mexico (Tlaltizapan), with the same protocol as described above. The design was an alpha lattice with three repetitions, and seven plants were measured per plot. Climatic conditions were recorded as above. The parameters for the model were obtained in the phenotyping platform PhenoArch, as presented in Welcker *et al.* (2011), except for N_final_ that was collected in the field.

## Results

### The genetic variability of the distributions of leaf length and width along leaf ranks primarily depended on flowering time

**Fig. 1.**
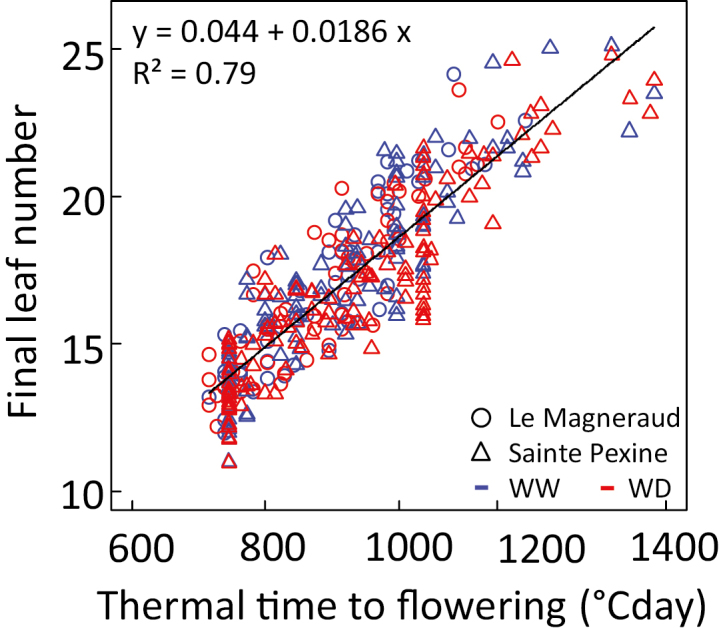
Relationship between the thermal time from emergence to flowering and the final leaf number for 119 maize lines in two field sites (Dataset B) under well-watered (blue dots) or water deficit (red dots) conditions. Black line: linear regression performed on the whole dataset.

The analysis of 119 maize lines (Dataset B) presenting the N_final_ from 12 to 25 was performed in two temperate field sites. This revealed that the effect of the genetic variability of time to flowering was adequately represented by that of N_final_ (Fig. 1, *R*^2^=0.79), whereas the time elapsed between the appearance of successive leaves (phyllochron) was independent of flowering time in the same diversity panel (Supplementary Fig. S2). Our ability to predict flowering time from leaf number was further tested by a 5-fold cross-validation. We randomly split the dataset of 119 lines grown in two conditions (238 data points) into five subsets (47–48 data points each). A regression equation between N_final_ and flowering time was calculated on the training set of 190 plants (four subsets), and the estimated parameters were used to predict flowering time of the test set (fifth subset). Prediction accuracy of flowering time was good, with a root mean square error (RMSE) of flowering time of 71.6 °Cd (equivalent to 4–5 d around flowering) and a coefficient (CV) of the RMSE of 7.8% (Supplementary Fig. S3). Hence, the genetic variability of flowering time was expressed as that of leaf number in further analyses.

**Fig. 2.**
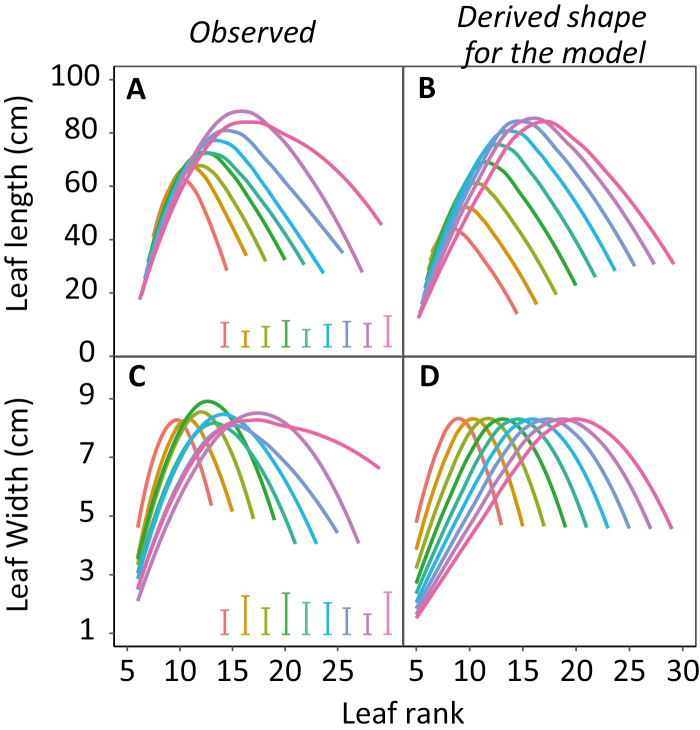
Distributions of final leaf length and width along leaf ranks for 127 maize lines with contrasting final leaf numbers, grown in five field experiments (dataset B). Each colour represents the average profile corresponding to lines with a common final leaf number in five sites. (A and C) Observed values; (B and D) distributions derived from the parameters B_L_ and G_L_ (Equation 11, Box 1) calculated from experimental data, for lines with each leaf number. Error bars show the average SDs for each group.

 We used the well-watered plots (five sites in France, Mexico, and Kenya) to analyse the effect of N_final_ on the distributions of leaf lengths and widths along leaf ranks (Fig. 2). The distribution of leaf length presented a common bell shape in all studied cases, with a maximum leaf length at about half of the N_final_ (Fig. 2A). The length of this longest leaf increased with N_final_. For example, the maximum length was 62 cm, on leaf 9, for plants presenting 14 leaves, whereas it was 88 cm, on leaf 16, for lines presenting 26 leaves. The leaves at ranks 1–10 were longer in the earliest hybrids (those presenting the smallest N_final_) than in the latest hybrids, but this did not compensate for the effect of the N_final_ on the cumulated leaf length per plant. The leaf with a maximum width was located at a rank slightly higher than that with maximum leaf length (Fig. 2C). Notably, the width of the widest leaf in a given plant, observed at a leaf rank that increased with N_final_, did not present a consistent relationship with N_final_, contrary to the case of leaf length.

### The time courses of leaf tip and ligule number were used for modelling leaf development

Modelling the expansion of individual leaves requires identification of the dates at which leaf elongation and widening begin and end, for each leaf rank on the stem of plants presenting different the final number of leaves. Direct observation of these dates involves daily dissection and measurement of all leaves that grow inside the plant whorl, an unfeasible task for hundreds of genotypes. Conversely, the dates of leaf tip and ligule appearance can be monitored for thousands of plants in a phenotyping platform and are consistent with those measured in the field (Millet *et al.*, 2019). Our model therefore considered the latter information to infer the timing of elongation and widening. We linked the dates of beginning of leaf elongation to those of leaf tip appearance, and the date of end of leaf elongation with those of ligule appearance, based on Dataset A (Table 1) involving two hybrids presenting contrasting flowering times and N_final_.

**Fig. 3.**
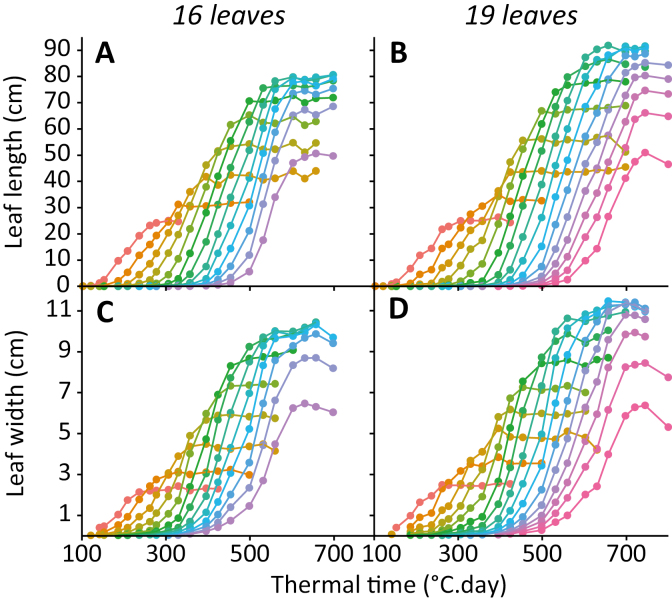
Time courses of leaf length and width for two maize hybrids with contrasting final leaf numbers (Déa, 16 leaves; Volga, 19 leaves, Dataset A, Table 1). Each colour is one leaf rank.

**Fig. 4.**
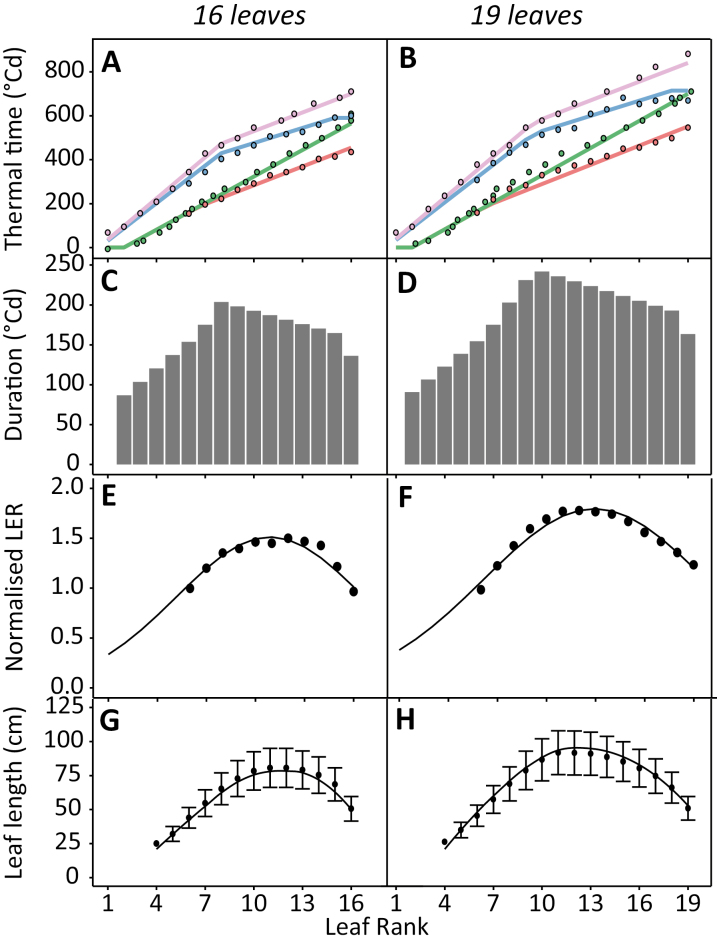
Thermal time to reach developmental stages (A, B), duration of leaf expansion (C, D), normalized leaf elongation rate (LER; E, F), and final leaf length (G, H) for two maize hybrids with different flowering time and final leaf number (Déa, 16 leaves; Volga, 19 leaves; Dataset A, Table 1). (A and B) Thermal time from emergence to beginning of linear expansion (red line), leaf tip appearance (green), end of linear expansion (blue), and ligule appearance (purple) as a function of leaf rank. Points, experimental data; lines, model. (E and F) Leaf elongation rate normalized by that of leaf 6. Each dot shows the mean value of 10 plants. The line is the fit with Equation 10. (G and H) Final leaf length. Each dot is the average of 10 plants ±SD. The line is the prediction by the model.

The time course of leaf length followed similar patterns for all leaves of the two studied genotypes (Fig. 3A, B), with a linear increase during most of the period of elongation, surrounded by periods of acceleration and deceleration. This linear increase was consistent with the constant LER measured in a phenotyping platform (Dignat *et al.*, 2013) and with the anatomy of the elongating zone in maize leaves (Tardieu *et al.*, 2000). For modelling purposes, we therefore approximated the time courses observed in Fig. 3A and B as three-domain linear curves (Supplementary Fig. S4). Leaf length was considered as null in a first domain, as equal to the maximum leaf length in the third domain, and to follow a linear increase in the intermediate domain. The dates for transition between these linear domains were calculated based on regressions of the progression of leaf length, with limits set at 5% and 95% of the final length of the leaf of the considered rank (*n*). We then related these dates to the appearance of leaf tips and ligules for every leaf rank on the stem. In both studied genotypes, leaf tips appeared sequentially (Fig. 4A, B) with a stable appearance rate. The thermal time from plant emergence to leaf tip appearance (*tt*_tip_) was therefore calculated from leaf rank *n* and the two genotypic parameters of the linear regression, namely the thermal time between subsequent leaf tip appearances (*a*_tip_) and the intercept of the regression (*b*_tip_; Box 1, Equation 1).

 The beginning of linear elongation occurred simultaneously, for the first leaves which appeared, with leaf tip appearance (Box 1, Equation 2) and then diverged at a common leaf rank for both genotypes (named *N*_bl_lim_ hereafter, considered non-genotypic and equal to 6)—when the tips of the sixth leaf appeared in the whorl (Fig. 4A, B). For leaves at ranks higher than *N*_bl_lim_, leaf tip appearance occurred after the beginning of linear elongation. The delay between these two events increased linearly with leaf rank, with a slope *k*_bl_ that was common to both hybrids, and considered as non-genotypic (Box 1, Equation 3).

The progress with time of the end of leaf elongation was approximated with a segmented-linear curve with two breakpoints (Fig. 4A, B). The first breakpoint was common to that of ligule appearance in both hybrids, at leaf rank 7 in the 16-leaf hybrid and leaf rank 9 for the 19-leaf hybrid (Fig. 4A, B). It was therefore simulated as (*n*=α _ll_*N*_final_), where α _ll_ is non-genotypic. Both the slope (α _ll1_) and intercept (*b*_ll1_) of the relationship between thermal time and ligule appearance at ranks lower than this breakpoint were considered as genotype dependent (Box 1, Equation 4). Because the ratio between the slopes before and after the breakpoint was common for both hybrids, we considered this ratio (*k*_ll_) as common to all hybrids (Box 1, Equation 5). The last two leaves (*N*_last_) stopped elongation simultaneously in both genotypes, thereby creating a second breakpoint in the progression of the end of leaf elongation. The thermal time from plant emergence to the end of linear elongation (*tt*_el_) of a leaf at rank *n* was modelled based on these observations (Box 1, Equations 6, 7).

The time course of leaf widening was similar to that of elongation but ending before it (Supplementary Fig. S4), as presented earlier (Lacube *et al.*, 2017). Hence, the beginning of widening (*tt*_beg,w_) was considered as common with that of elongation, and the end of widening (*tt*_end,w_) was considered to occur 39 °Cd before that of elongation (lag_w_ 39 °Cd; non-genotypic, Lacube *et al.*, 2017; Box 1, Equations 7, 8).

Overall, five genotypic parameters needed to be measured for the simulation of the timing of the growth of all leaves of a plant, namely the N_final_, the parameters of the regressions between thermal time and leaf tip appearance (a_tip_ and b_tip_), and those between thermal time and ligule appearance (a_ll1_ and b_ll1_). The thermal time between the beginning and end of leaf elongation of each leaf defined the duration of leaf elongation (Fig. 4C, D).

### The distribution of maximum leaf elongation rate along the stem was modelled with genotypic and environmental effects

LER at a given time depends on a maximum rate, itself depending on the considered leaf rank and genotype, and environmental effects (Welcker *et al.*, 2011; Lacube *et al.*, 2017).

The genotypic effect was taken into account via one parameter per genotype, the maximum LER of leaf 6 that can be measured on a phenotyping platform (Welcker *et al.*, 2011). This rate ranged from 4.07 mm °Cd^–1^ to 6.17 mm °Cd^–1^ in the diversity panel of Dataset B (Supplementary Table S4), and showed a clear genetic correlation with final leaf length (Dignat *et al.*, 2013).

The effect of leaf rank was analysed (Fig. 4E, F) for hybrids showing different N_final_. The maximum LER (LER_max_ expressed per unit of thermal time) was calculated for each leaf rank based on its time course (Fig. 3; Supplementary Fig. S4). It was then normalized (LER_norm_) by the maximum rate of leaf 6 (a_6_) of the considered genotype. In both studied hybrids, the distribution of normalized rates along leaf ranks showed a bell-shaped curve (Fig. 4E, F), fitted with a beta function with three parameters: θ _L_, the maximum relative LER; B_L_, the rank of the leaf with the highest growth rate; and G_L_, the curvature of the curve, all depending on N_final_ (Box 1, Equation 11).

The resulting distribution of leaf length along leaf ranks was obtained for the two hybrids in Fig. 4G and H. The two parameters driving the shape (B_L_ and G_L_, Equation 11 in Box 1) were considered as non-genotypic and dependent on N_final_ only. This was tested with the distributions of leaf length for genotypes differing in N_final_, presented in Fig. 2A. Considering B_L_ and G_L_ as linearly related to N_final_ allowed us to reproduce the shapes of the observed distributions (Fig. 2B). This was achieved only if the LER of leaf 6 decreased with the duration from emergence to flowering time, confirming a tendency observed in previous studies (Dignat *et al.*, 2013).

### The actual leaf elongation rate was modelled based on environmental conditions during the growth of the considered leaf cohort

Although a model taking into account the temporal variation of water potentials along the plant and circadian rhythms provides insights into the diurnal variations of LER (Caldeira *et al.*, 2014; Tardieu *et al.*, 2015), it cannot be used at this stage for a large number of hybrids because of the number of parameters and the complexity of their measurements with current phenotyping methods. Hence, we kept a formalism in which the time course of the leaf elongation rate is modelled by considering regressions of the LER with temperature, evaporative demand, and soil water potential (Welcker *et al.*, 2011). Notably, light was taken into account only via its effects on meristem temperature and evaporative demand because we found no indication for a direct effect (Salah and Tardieu, 1996; Lacube *et al.*, 2017). During the period of elongation (from *tt*_bl_ to *tt*_el_), the elongation rate of any growing leaf during day *d* was then calculated from that of leaf 6, the distribution of the normalized LER along leaf ranks, and the effects of evaporative demand and soil water potential (Box 1, Equation 12).

### The distribution of leaf width along leaf ranks was modelled with genotypic and environmental effects

In the same way as for leaf length, we considered a reference distribution of leaf width (W_base_) along leaf ranks, which depends on genotype and leaf rank, and an environmental effect that depends on intercepted light (RAD_effect_) (Box 1, Equation 13).

The genotypic effect was taken into account via the width of leaf 6 (W_6_), which can be measured on a phenotyping platform (Lacube *et al.*, 2017), and ranged from 66 mm to 83 mm in a panel of hybrids (dataset C). 

**Fig. 5.**
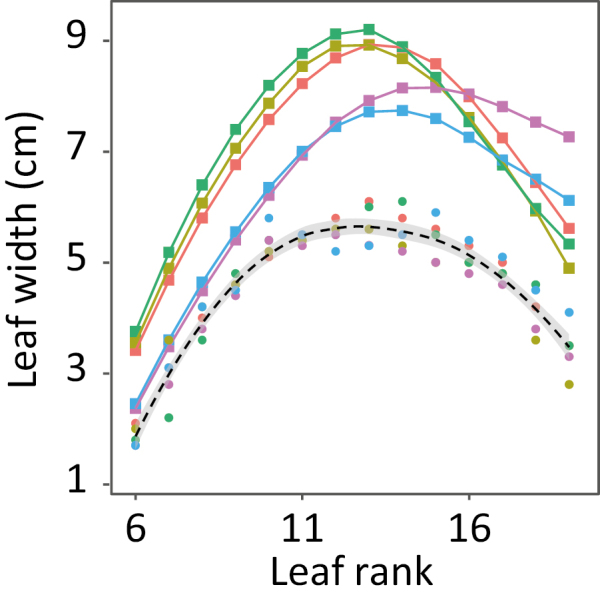
Averaged profiles of final leaf width for lines presenting a final leaf number of 19 in five field experiments with contrasting light intensity (Dataset B, Table 1). Squares, observed values measured in Mauguio (red), Nerac (light green), Le Magneraud (green), Tlaltizapan (blue), and Kiboko (pink). Circles represent the calculated width for an intercepted light of 1.5 MJ m^–2^ d^–1^ during the period of widening of the corresponding leaf; that is, removing the effect of intercepted light from observed data. Dashed line, overall profile fitted by the smoothing function loess() of R (span=0.65). Grey shading shows the confidence interval.

The reference distribution W_base_ was calculated based on the response of leaf width to intercepted radiation (genotypic parameter r_RAD_, the sensitivity of leaf widening to intercepted radiation, Box 1, Equation 14). Indeed, leaf width has a strong positive sensitivity to whole-plant intercepted radiation (RAD_i_) but no response to evaporative demand (Lacube *et al.*, 2017). For the two hybrids of Dataset A, we first calculated the reference distribution of width along leaf ranks by removing the effect of intercepted radiation (Supplementary Fig. S5). The same was performed on genotypes of Dataset B (Fig. 5), resulting in a common distribution of leaf width for the five experiments of the dataset. Indeed, measured leaf widths greatly differed between experiments, but differences were accounted for by the intercepted radiation. Notably, the distributions of leaf width were obtained in experiments carried out in France, Mexico, and Kenya, suggesting a wide relevance of the mechanism of control of leaf width presented in Lacube *et al.* (2017). Figure 5 also shows that the distribution of leaf width can be considered as a stable trait of a given genotype. Then, these distributions were formalized by a beta function (Box 1, Equation 15), similar to that used for the LER_max_, with parameters depending on N_final_ and a genotype-specific effect considered via the reference width of leaf 6 (W_6_).

Overall, our model considered only two genotypic parameters to calculate the widths of individual leaves, namely the sensitivity to intercepted light and the reference width of leaf 6. These parameter values can be inferred from measurements of one leaf in two light conditions. The model was used to simulate the distributions of leaf width along leaf ranks, observed for genotypes with N_final_ ranging from 13 to 29 (Fig. 2C). As for leaf length, considering B_w_ and G_w_ (Equation 15, Box 1) as linearly related to N_final_ allowed us to reproduce the shapes of the observed distributions (Fig. 2D).

### The resulting model accounted for genotypic and environmental effects in independent datasets

**Fig. 6.**
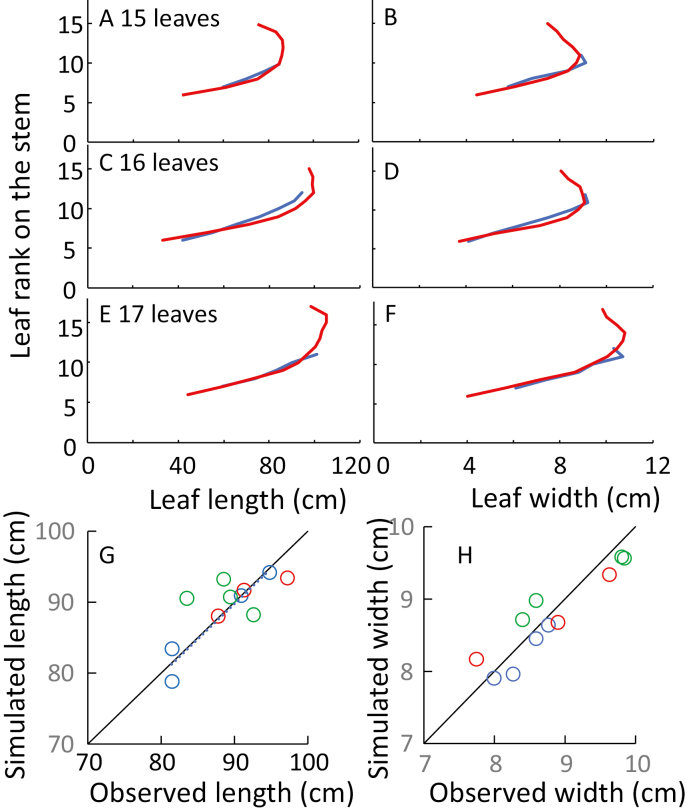
Distributions of leaf length and width along leaf ranks on the stem, and observed versus simulated leaf length and width. (A, C, and E) Leaf length, simulated (red) or measured (blue); (B, D, and F) leaf width. (A and B) Hybrid F252_H with a final leaf number of 15; (C and D) hybrid B73×UH007 with a final leaf number of 16; (E and F) hybrid B104_H with a final leaf number of 17. (G) Observed versus simulated lengths for all hybrids, mean values for leaves 9–11. (H) Observed versus simulated widths, mean values for leaves 9–11. In (G) and (H), blue, green, and red indicate plants with a final leaf number of 15, 16, and 17, respectively; black lines are the 1:1 line. In (G), *r*^2^=0.58; in (H), *r*_2_=0.87.

The model was first tested on final leaf length and width of 14 hybrids, with N_final_ ranging from 15 to 17 (Fig. 6, Dataset F). The 10 genotypic parameters were obtained for each hybrid (i) in the platform PhenoArch (Dataset C, Table 1) for the slopes and intercepts of the relationship of leaf appearance and ligulation with thermal time (a_tip_, b_tip_, a_ll1_, and b_ll1_), LER_max_ of leaf 6 (a_6_), and sensitivities of leaf elongation to evaporative demand (b) and to soil water deficit (c); (ii) in the field Dataset D for the N_final_, the reference width of leaf 6 (W_6_); and (iii) in Lacube *et al.*, (2017) for the leaf widening sensitivity to plant intercepted radiation (r_RAD_), which was considered here as genotype independent. Parameter values are summarized in Supplementary Table S4. The same protocol was applied to 17 lines of the population P1P2 (Welcker *et al.*, 2007; Dataset G) for which leaf length along leaf ranks and N_final_ were measured in a field in Mexico, with parameters a, b, and c collected in the platform Phenodyn (Welcker *et al.*, 2011).

The distributions of leaf length and width were similar for observed and simulated data. As in Dataset B, the length of the longest leaf increased with N_final_ and was located at a higher rank for the latest genotypes. The same tendency was observed, for N_final_ from 19 to 23, in the experiment in Mexico (Supplementary Fig. S6). It was not observed for leaf width, consistent with the shapes presented in Fig. 2. Simulated leaf length and width averaged for leaves 9–11 accounted for observed length and width at the same ranks in Dataset F (*r*^2^=0.58 and 0.87 for length and width, respectively). The same was observed for the experiment in Dataset G for leaves 15–17. Notably, the regression applied to hybrids with different N_final_. This was due to genotypic differences in sensitivity measured in the greenhouse, which accounted for genotypic differences in the field.

We then considered the leaf length and width of plants of the reference hybrid B73×UH007, measured in 14 field experiments under well-watered conditions but contrasting evaporative demand and light (Dataset E, Table 1). The environmental conditions sensed by plants during leaf growth resulted in appreciable differences in measured leaf lengths along the stem, but less so for leaf width (Supplementary Fig. S7). The mean length of ranks 9–11 across sites was adequately simulated (*r*^2^=0.66). The same applied to leaf width, with a looser correlation (*r*^2^=0.44).

## Discussion

Process-based crop simulation models help the decision for crop×environment×management interaction (Muller and Martre, 2019), thereby requiring simulation of hundreds of genotypes in thousands of environment×management scenarios under present and future conditions (Parent *et al.*, 2018). This requires a dialogue between model formalisms and phenomic methods (Wang *et al.*, 2019) for measuring genotype-dependent model parameters of hundreds of genotypes in phenotyping facilities (Tardieu *et al.*, 2017). This study applies this approach.

The rules for simulating leaf growth in a wide genetic diversity of flowering time and environmental conditions were established based on experimental data, rather than on theoretical laws that are not available at this scale of plant organization. As presented previously, we considered as ‘metamechanisms’ those response curves to environmental conditions or developmental relationships which are valid across a large range of conditions, involving both indoor and field conditions (Tardieu and Parent, 2017; Tardieu *et al.*, 2018). This required the use of four datasets: (i) five field experiments with 127 maize lines showing contrasting leaf numbers; (ii) one field experiment with lines with higher N_final_; (iii) one experiment in a phenotyping platform; and (iv) one low-throughput field experiment with two genotypes for detailed time courses of leaf length and width. On one hand, it can be considered that the model presented here is based on a considerable amount of data for both establishment and testing of the model. However, we acknowledge that some of the rules proposed here are fragile, in particular (i) for the decisions as to whether a particular parameter is genotype dependent or can be considered as valid at the whole-species level; (ii) for the rules about synchronisms of leaf stages with the timing of elongation of individual leaves; and (iii) the model was partly established on lines and tested on hybrids. Another feature of the model is that we relied on a dynamic approach for leaf elongation, based on previous work of Welcker *et al.* (2011), but on a statistical approach for leaf width (Lacube *et al.*, 2017). In spite of these shortcuts, this model is the first,to our knowledge, to allow simulatation of the leaf dimensions of all leaves of a wide range of genotypes under contrasting environmental conditions (Tardieu and Parent, 2017). The simplification of Lizaso *et al.* (2003), who considered the dimension and rank of the largest leaf for simulations, is efficient, but cannot be used unless measurements are carried out in every field and every genotype to be simulated. In contrast, we show here that our model is robust enough to apply to new fields, based on 10 generic parameters that can be considered as genotype dependent (i.e. independent from the environment in which the model is used) and can be measured at high throughput in a phenotyping platform.

The limited number of genotypic parameters was a necessary condition for designing a model in which parameters are directly estimated via measurements of traits presenting a short ‘phenotypic distance’ with parameters (Tardieu *et al.*, 2018). Limiting the number of parameters was based on different arguments. (i) It was, in some cases, based on experimental results. This is the case for the response of growth and development processes to temperature, common to genotypes with diverse origins, in particular either tropical or temperate (Parent and Tardieu, 2012). (ii) In other cases, it was based on an experimentally established genetic correlation, as in the case of the sensitivities of leaf elongation rate to soil water deficit and evaporative demand (Welcker *et al.*, 2011). (iii) In many cases, it was a deliberate exercise, for example in the relationship between the genotypic leaf number and the parameters controlling the distribution of leaf width or length along leaf ranks. (iv) Finally, we acknowledge that in some case this choice was linked to the absence of available data, so the parsimony principle led us to consider that parameters did not differ between genotypes.

It is probably useful to re-affirm here that the purpose of this model was not to improve predictions for a standard genotype. If this was the case, a model based on a big leaf approach is more parsimonious. Indeed, the improved generality of our model will not mechanically translate to more accurate predictions of leaf area for one genotype. Our aims were here (i) to better analyse and simulate the genotype×environment interaction for leaf area. In particular, we used a preliminary version of this model to simulate the consequences of climate change on yield if farmers made the best possible use of the diversity of flowering time in each field in current and future conditions (Parent *et al.*, 2018). (ii) A second possible use of this model is to simulate the consequences of plant architecture traits on light interception. We have recently shown that changes in distribution of leaf area along leaf ranks allowed a genetic progress of the amount of light intercepted by the canopy layer hosting maize ears (Perez *et al.*, 2019). Finally, the 10 genotype-dependent parameters of the model may be predicted via genomic prediction based on a set of platform experiments such as those in Dataset C, thereby allowing one to predict the leaf area of hundreds of genotypes from their genotypic markers in hundreds of fields, as recently carried out for flowering time (Li *et al.*, 2018) or for the responses of yield to environmental conditions (Millet *et al.*, 2019).

## Supplementary data

Supplementary data are available at *JXB* online.

**Fig. S1**. Sensitivity of leaf elongation rate to soil water potential at the single-leaf level (Phenodyn platform) and sensitivity of the leaf expansion rate at the whole-plant level (PhenoArch platform).

**Fig. S2**. Relationship between the thermal time from emergence to flowering and the final leaf number or the phyllochron for 114 maize lines in two field sites (Dataset B).

**Fig. S3**. Cross-validation of prediction of flowering time from final leaf number.

**Fig. S4**. Time courses of leaf length and width of leaf 6 for two maize hybrids with different final leaf numbers (Dataset A).

**Fig. S5**. Profiles of leaf width for two maize hybrids with different final leaf number (Dataset A).

**Fig. S6**. Distributions of leaf length along leaf ranks on the stem, observed versus simulated in 17 hybrids (Dataset G, Mexico).

**Fig. S7**. Observed and simulated distributions of final leaf length and width for the maize hybrid B73×UH007, in 14 field experiments (with contrasting evaporative demand and light intensities (Dataset E).

**Table S1**. Summary of field trials of Dataset B.

**Table S2.** Presence of each genotype in each field trial for Dataset B.

**Table S3**. Summary of experiments used for model parameterization and validation.

**Table S4**. Parameter values for the 14 hybrids used in model validation.

eraa278_suppl_Supplementary_Material
