# Supplementary material for: Simulating the effect of flowering time on maize individual leaf area in contrasting environmental scenarios
Source: J Exp Bot. 2020 Jun 11;71(18):5577–88. doi: 10.1093/jxb/eraa278 (PMC7501815; doi:10.1093/jxb/eraa278)
Supplement: eraa278_suppl_Supplementary_Material [file eraa278_suppl_supplementary_material.pdf]

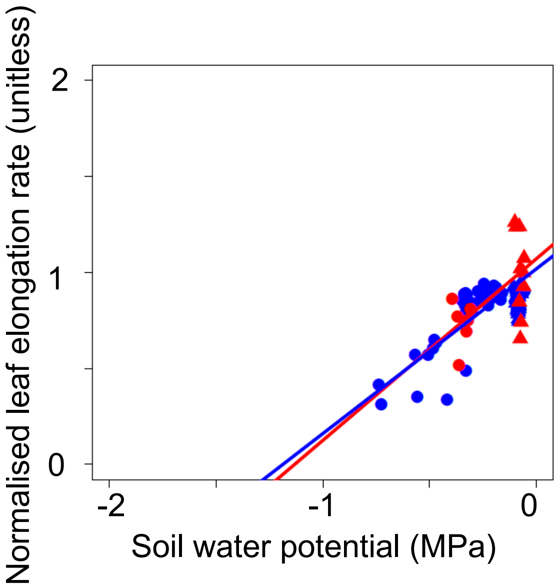

Supplementary Figure S1. Sensitivity of leaf elongation rate to soil water potential at single leaf level (Phenodyn platform, red) or sensitivity of leaf expansion rate at whole plant level (PhenoArch platform, blue). Both were normalized by the maximum value, in well watered soil.

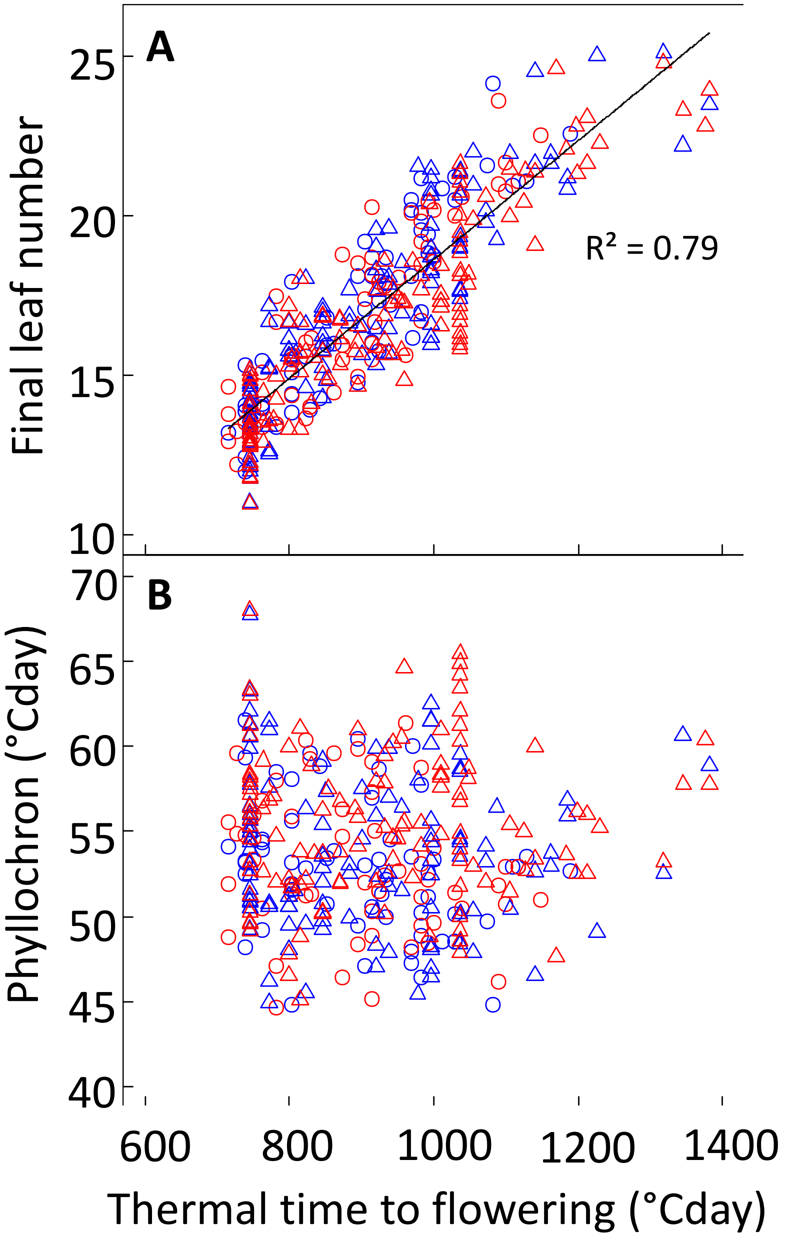

Supplementary Figure S2. Relationship between the thermal time from emergence to flowering and (A) the final leaf number or (B) the phyllochron for 114 maize lines in two field sites (circles : Le Magneraud; triangles : Sainte Pexine, Dataset B) under well watered (blue dots) or water deficit (red dots). A. Black line, linear regression performed on the whole dataset (Final leaf number = 0.0186 thermal time + 0.044).

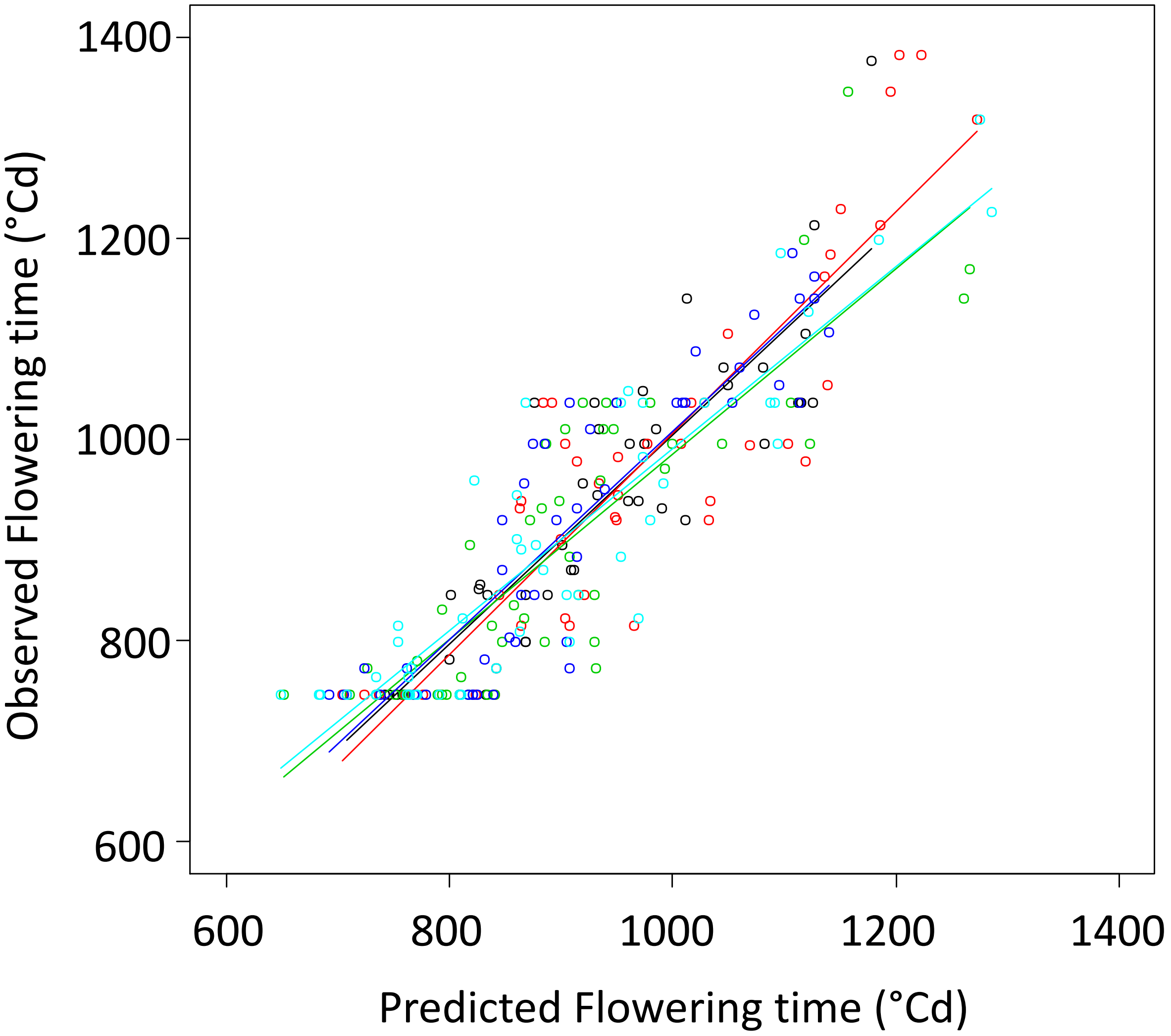

Supplementary Figure S3. Cross validation of prediction of flowering time from final leaf number. The full dataset of Figure 1 (119 genotypes grown in two watering conditions) was randomly subdivided into five subsets comprising 23-24 genotypes in well watered and water deficit conditions. Flowering time in each subset was predicted from the linear regression between final leaf number and flowering time, whose equation was obtained based on the other four other subsets. Dots display predicted and observed values and lines display their linear regression in each subset (five different colors for five subsets).

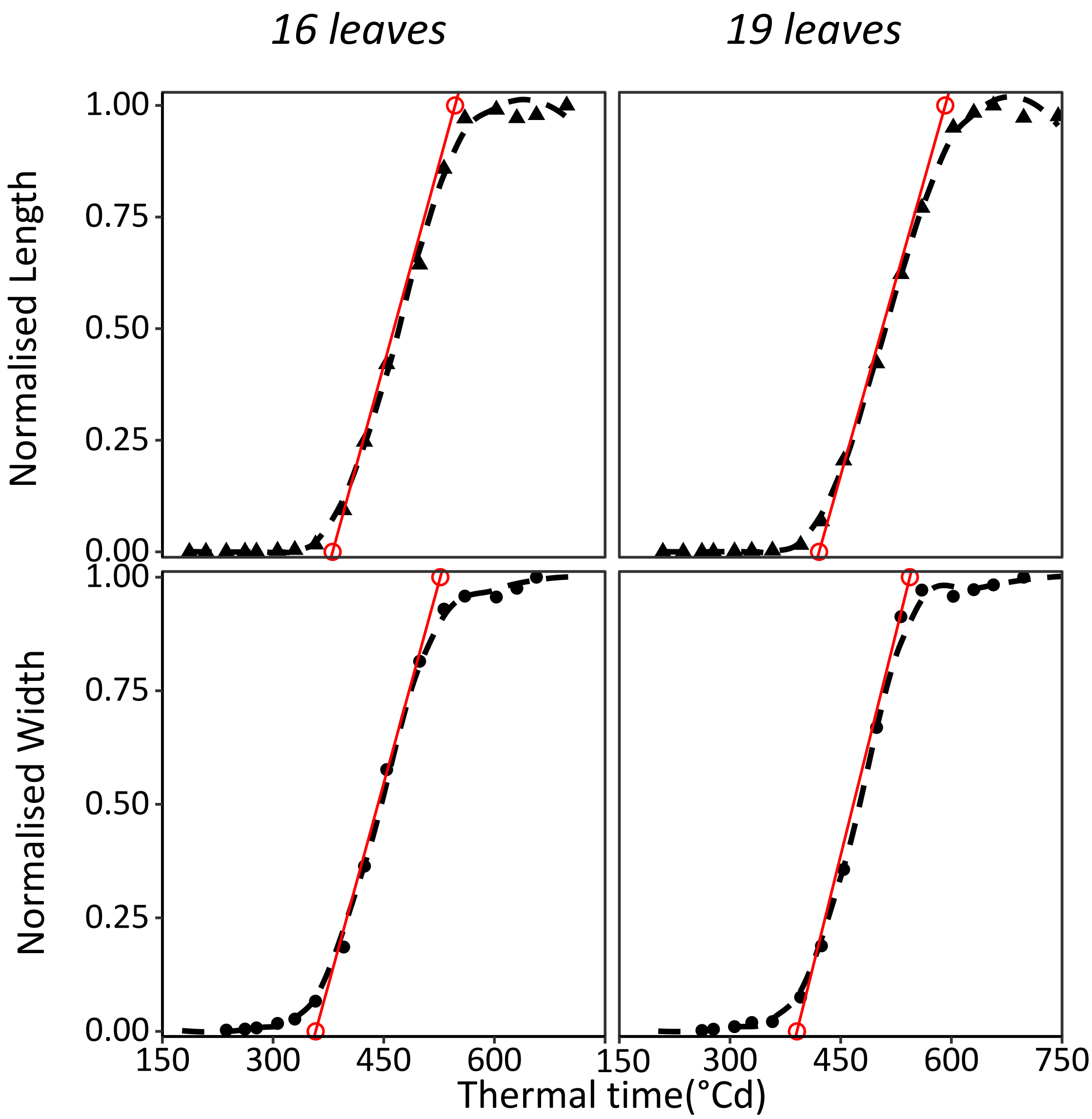

Supplementary Figure S4. Time courses of leaf length and width of leaf 6 for two maize hybrids with different final leaf numbers (dataset A, Table 1). Black dots, observed values (n= 10) measured destructively every second day. Black dashed curves are fitted with a smoothing function loess() in R (Span = 0.5). Red lines are the linear models fitted between the begining and end of elongation and widening, estimated from smoothed curves as the 5% and 95% of final dimensions.

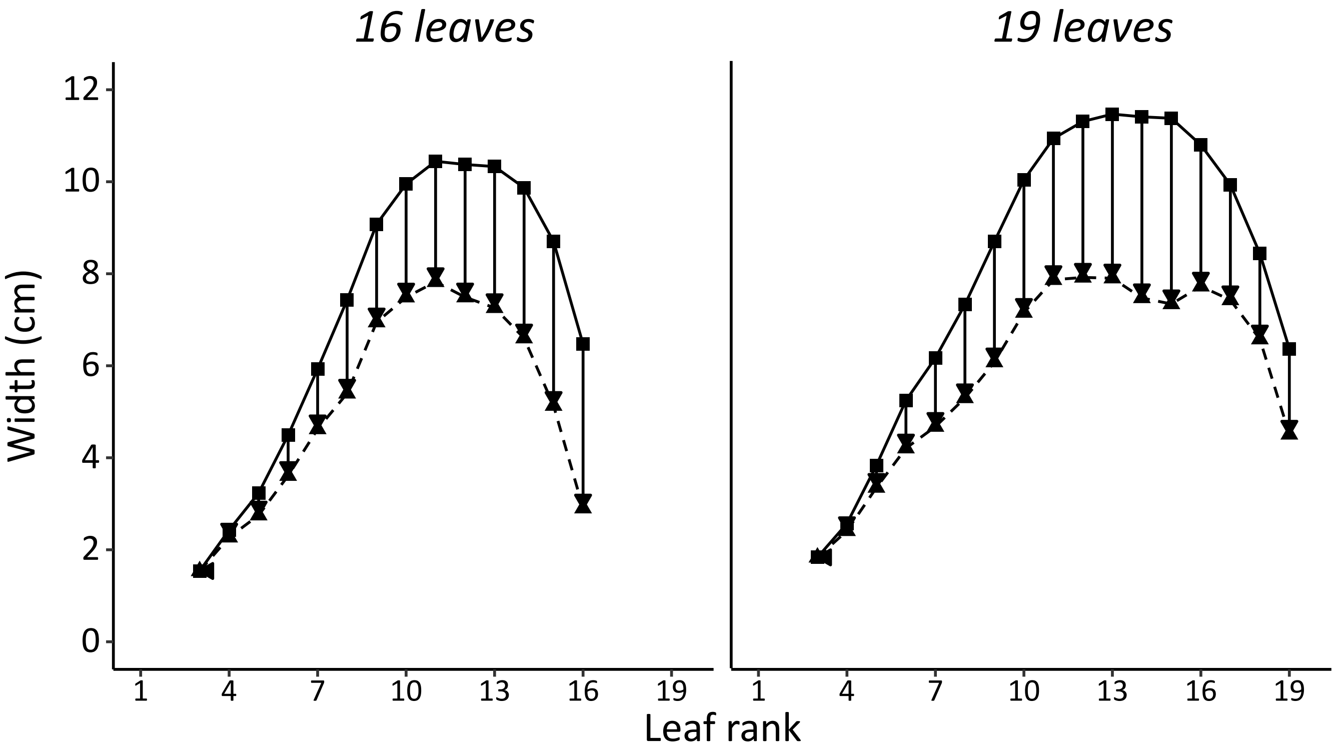

Supplementary Figure S5. Profiles of leaf width for two maize hybrids with different final leaf numbers (dataset A, Table 1). Squares, observed values (n= 10). Triangles, calculated width for a intercepted light of 1.5 MJ m^{-2} d^{-1} during the period of widening of the corresponding leaf, i.e. removing the effect of intercepted light from observed data.

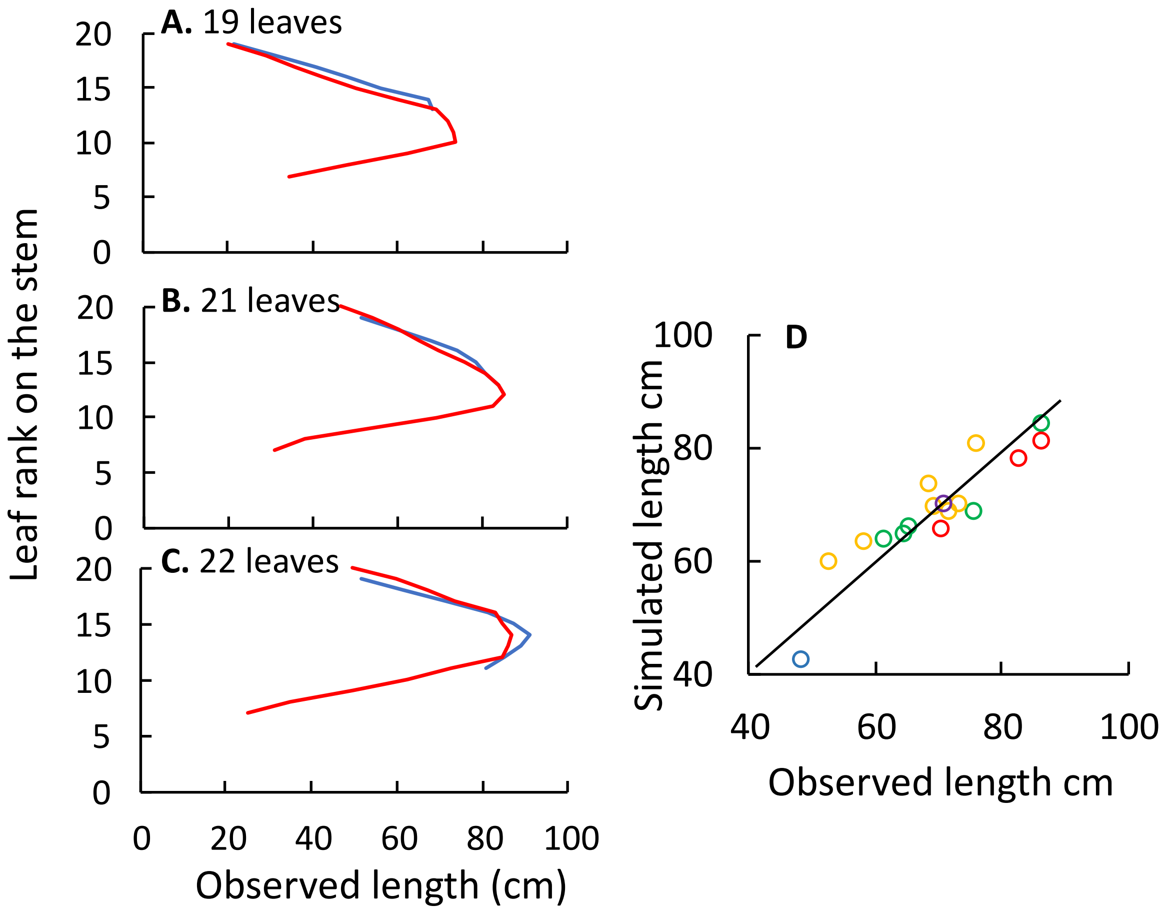

Supplementary Fig S6. Distributions of leaf length along leaf ranks on the stem, observed (blue) vs simulated (red) in dataset G (Mexico). A, line 214 with a final leaf number of 19; B, line 114 with a final leaf number of 21; C, line 35 with a final leaf number of 22. D, observed vs simulated lengths for 17 hybrids, mean values for leaves 15-17; blue, green orange, red and purple dots, plants with a final leaf number of 19, 20, 21 22 and 23, respectively; black line, 1:1 line. $r^2 = 0.83$. Model parameters for each line are below.

Genotype	Model parameters			
	a_6 mm.°Cd^{-1}	b mm.°Cd^{-1}.kPa^{-1}	c mm.°Cd^{-1}.MPa^{-1}	N_{final} leaf
10	4.69	-1.28	4.16	22
25	4.22	-1.08	4.48	21
38	3.99	-0.96	4.65	21
40	3.58	-1.01	3.19	22
63	4.83	-1.49	5.05	20
82	3.95	-0.96	3.60	20
95	4.04	-1.13	4.32	21
114	3.09	-0.90	2.72	21
119	3.52	-1.00	3.42	21
123	4.10	-1.30	4.87	23
139	3.27	-0.89	4.11	20
156	4.60	-1.38	4.61	20
170	4.08	-0.90	5.02	21
177	3.79	-1.19	3.75	20
208	3.81	-0.84	4.06	22
214	3.65	-0.98	3.80	19
245	4.75	-1.12	4.95	21

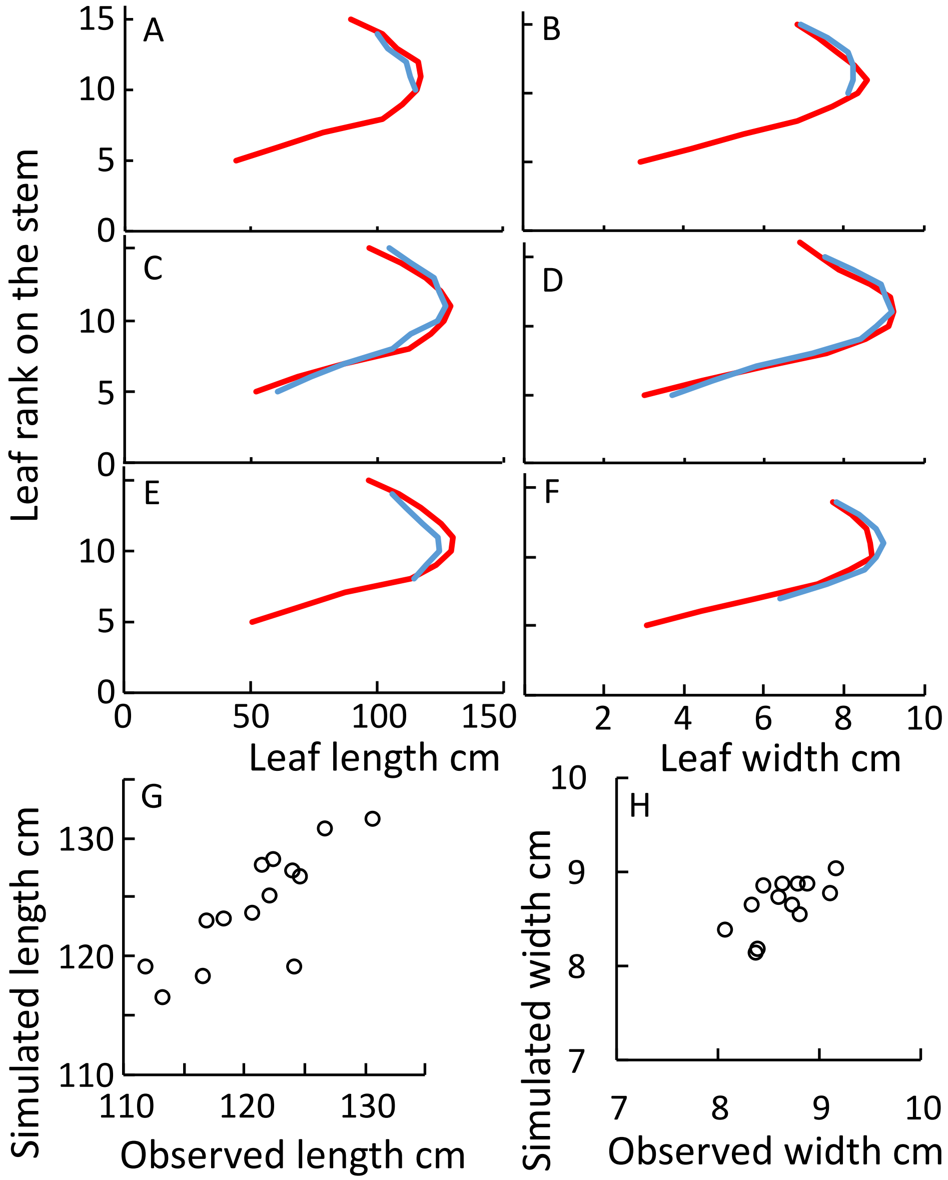

Supplementary Figure S7. Observed (red) and simulated (blue) distributions of final leaf length and width for the maize hybrid B73 x UH007, in 14 field experiments (Dataset E, Table1, details in SI Table S2) with contrasting evaporative demand and light intensities (SI Table S2) in Caracal 2012 (A,B), Mauguio 2011 (C,D) and Karlsruhe 2013. (E,F) G, observed vs simulated lengths for all sites, mean values for leaves 9-11; H observed vs simulated widths, mean values for leaves 9-11. In G, r^2=0.66; in H r^2 = 0.44.

Supplementary Table S1 . Summary of field trials of dataset B

Site	Year	ID	Latitude (°N)	Longitude (°E)	Altitude (m)	T_{max} (°C)	T_{min} (°C)	T_{mean} (°C)	VPD_{max} (kPa)	RAD_{sum} (MJ/m²)	Number of genotypes	Precocity range
Kiboko, Kenya	2007	KI07p	2,3	37,8	915	24,2	11,3	17,7	1,1	2958	6	19 - 21
Le Magneraud, France	2007	LM07	46,8	0,75	25	21,5	12,1	16,8	1,2	2717	56	12 - 25
Mauguio, France	2007	MA07	43,61	3,98	13	24,7	15,4	20,0	1,7	3231	104	12 - 16
Nerac, France	2006	NR06	44,17	0,31	40	21,4	13,2	17,3	1,6	2788	117	14 - 29
Tlaltizapan, Mexico	2007	TL07p	18,41	99,1	940	29,3	16,8	23,1	1,2	2142	6	19 - 21

All environmental variables are calculated in the period between sowing and ligulation of the last leaf for the reference genotype B73_H. T_{max} : Mean of daily maximum temperature. T_{min} : mean of daily minimum temperature. Tmean : mean of T_{max} and T_{min} . VPD_{max} : mean of daily maximum vapor pressure deficit. RAD_{sum} : sum of daily radiation.

Supplementary Table S2. Presence of each genotype in each field trial for dataset B.

Genotype	Origin*	KI07p	LM07	MA07	NR06	TL07p
K64R	S-Trop	X	X			X
CML245	S-Trop	X		X		X
CML247	Trop	X				X
KUI3	Trop	X				X
H16	Trop	X				X
SCMALAWI	S-Trop	X			X	X
F2834T	Temp		X		X	
B73-t	Temp		X		X	
L256	Temp		X	X	X	
CI1872U	Temp		X		X	
NC298	Temp		X		X	
KY21	Temp		X		X	
NC304	Temp		X		X	
NC320	Temp		X		X	
HP301	Temp		X	X	X	
OH43	Temp		X			
B73	Temp		X	X		
MO17	Temp		X	X	X	
N25	Temp		X	X	X	
LP35	Temp		X	X	X	
F64	Temp		X	X	X	
ZN6	Temp		X	X	X	
MBS-t	Temp		X			
LAN496	Temp		X	X	X	
LMGC47	Temp		X	X	X	
LMGC12	Temp		X	X	X	
EA1712	Temp		X	X	X	
N6	Temp		X	X	X	
EA1433	Temp		X	X	X	
EA1201	Temp		X	X	X	
RAGT113	Temp		X	X	X	
MBS847	Temp		X	X		
FC25	Temp		X	X	X	
NY302	Temp		X	X	X	
F591	Temp		X	X	X	
FC209	Temp		X	X	X	
PB40R	Temp		X	X	X	
LO3	Temp		X	X	X	
EA1174	Temp		X	X	X	
BA90	Temp		X	X	X	
F39	Temp		X	X	X	
F471	Temp		X	X	X	
CH10	Temp		X	X	X	
LMGC90	Temp		X	X	X	
FC1852	Temp		X	X	X	
FV252	Temp		X		X	
FC24	Temp		X	X	X	
YUBR5	Temp		X	X	X	
W117U	Temp		X	X	X	
FC16	Temp		X	X	X	
FV65	Temp		X	X	X	
FV76	Temp		X	X	X	
W85	Temp		X	X	X	
FV2	Temp		X	X	X	
FV71	Temp		X	X	X	
EP1	Temp		X	X	X	
FV75	Temp		X	X	X	
F252-t	Temp		X		X	
ND30	Temp		X	X	X	
RAGT175	Temp		X	X	X	
FV7	Temp		X	X	X	
F252	Temp			X	X	
B106	Temp			X	X	
P9C0S6	Temp			X	X	
PLS41	Temp			X	X	
ND36	Temp			X	X	
PA374	Temp			X	X	
C105	Temp			X	X	
F7001	Temp			X	X	
FC42	Temp			X	X	
A374	Temp			X	X	
RAGT84	Temp			X	X	
LMGC28	Temp			X	X	
EA1070	Temp			X	X	
LMGC93	Temp			X	X	
RAGT127	Temp			X	X	
B104U	Temp			X	X	
MO15W	Temp			X	X	
Oh43	Temp			X	X	
RAGT3	Temp			X	X	
F48	Temp			X	X	
FV230	Temp			X	X	
PA36	Temp			X	X	
LMGC80	Temp			X	X	
ND283	Temp			X	X	
F712	Temp			X	X	
W103	Temp			X	X	
FV344	Temp			X	X	
FV232	Temp			X	X	
FV72	Temp			X	X	
EA1301	Temp			X	X	
PLS14	Temp			X	X	
CO158	Temp			X	X	
WJ	Temp			X	X	
LMGC91	Temp			X	X	
F7038	Temp			X	X	
FV4	Temp			X	X	
F7012	Temp			X	X	
CL18	Temp			X	X	
RAGT67	Temp			X	X	
H99USA	Temp			X	X	
PB57	Temp			X	X	
F608	Temp			X	X	
RAGT185	Temp			X	X	
P465P	Temp			X	X	
Oh33	Temp			X	X	
Oh40B	Temp			X	X	
RAGT4	Temp			X	X	
LMGC88	Temp			X	X	
F759	Temp			X	X	
LO33	Temp			X	X	
F473	Temp			X	X	
PLS6	Temp			X	X	
RAGT12	Temp			X	X	
FV324	Temp			X	X	
ND1	Temp			X	X	
LMGC92	Temp			X	X	
CQ191	Temp			X	X	
FV74	Temp			X	X	
LMGC89	Temp			X	X	
EA1866	Temp				X	
MBS847-t	Temp				X	
RAGT189	Temp				X	
TZI18	Trop				X	
GT112U	Trop				X	
MO22	Temp				X	
A6	Temp				X	

* Trop : Tropical genotype. Sub-trop : sub-tropical genotype. Temp : temperate genotype.

Supplementary Table S3. Summary of experiments used for model parameterisation and validation

Dataset	Location	Country	Year	ID	Latitude (°N)	Longitude (°E)	Altitude (m)	Sowing date (day)	Density ($plants.m^{-2}$)	T_{max} (°C)	T_{min} (°C)	T_{mean} (°C)	VPD_{max} (kPa)	RAD_{sum} (MJ/m²)
C	Montpellier (PhenoArch platform)	France	2016	Mon16	43,62	3,86	43	10/05/2016	11,0	25,1	18,4	21,7	1,0	1155
D	Saint Martin	France	2016	StM16	43,57	-1,3	4	17/05/2016	10,0	22,3	12,8	17,5	2,1	2359
E	Cadriano	Italy	2012	Cad12	44,93	11,88	5	17/05/2012	6,3	24,3	15,7	20,0	1,8	3675
	Campagnola	Italy	2011	Cam11	45,17	9,53	49	10/05/2011	6,4	23,2	15,1	19,1	1,4	3120
	Campagnola	Italy	2013	Cam13	45,17	9,53	49	14/06/2013	5,7	22,8	13,8	18,3	1,9	2328
	Caracal	Romania	2012	Car12	44,12	24,35	101	21/05/2012	6,7	28,7	14,2	21,4	1,8	3315
	Karcag	Hungary	2011	Karc11	47,18	20,54	81	13/05/2011	6,7	23,6	11,7	17,6	2,0	3060
	Karcag	Hungary	2012	Karc12	47,18	20,54	81	21/05/2012	5,7	24,3	11,8	18,0	1,7	2820
	Karlsruhe	Germany	2011	Karl11	49,07	8,28	115	04/05/2011	8,2	20,5	10,0	15,2	1,5	2595
	Karlsruhe	Germany	2012	Karl12	49,07	8,28	115	11/05/2012	8,2	19,8	10,1	14,9	1,4	2520
	Karlsruhe	Germany	2013	Karl13	49,07	8,28	100	20/05/2013	8,2	18,1	10,2	14,1	1,3	2385
	Mauguio	France	2011	Mau11	43,61	3,98	13	14/05/2011	7,5	23,4	14,2	18,8	1,6	3105
	Murony	Hungary	2013	Mur13	46,78	21,05	85	22/05/2013	6,7	22,6	11,1	16,9	2,1	2715
	Nerac	France	2011	Ner11	44,17	0,31	40	13/05/2011	6,9	25,0	11,3	18,2	1,8	2910
	Nerac	France	2012	Ner12	44,17	0,31	40	28/05/2012	7,4	26,4	10,1	18,3	1,5	3000
	Nerac	France	2013	Ner13	44,17	0,31	40	15/05/2013	7,4	24,0	10,5	17,3	1,7	2850
F	Mauguio	France	2016	Mau16	43,61	3,98	13	16/05/2016	7,2	23,2	14,3	18,8	1,3	3105

All environmental variables are calculated in the period between sowing and ligulation of the last leaf for the reference genotype B73_H. T_{max} : Mean of daily maximum temperature. T_{min} : mean of daily minimum temperature. Tmean : mean of T_{max} and T_{min} . VPD_{max} : mean of daily maximum vapor pressure deficit. RAD_{sum} : sum of daily radiation.

Supplementary Table S4. Parameter values for the 14 hybrids used in model validation

Genotype	Measured model parameters									
	Platform							*Field*		*Field & Platform*
	a_{tip}	b_{tip}	a_{ll1}	b_{ll1}	a_6	b	c	N_{final}	W_6	r_{RAD}
	°Cd $leaf^{-1}$	°Cd	°Cd $ligule^{-1}$	°Cd	mm $°Cd^{-1}$	mm $°Cd^{-1}$ kPa^{-1}	mm $°Cd^{-1}$ MPa^{-1}	leaf	mm	mm $°MJ^{-1}$
B104_H	42	-64	67	125	3.57	-1.36	5.01	17	49	19
B73_H	51	-49	86	137	3.19	-1.17	3.52	16	41	34
B97_H	47	-63	72	132	3.93	-1.51	6.19	16	58	28
EC136_H	43	-56	66	123	3.45	-1.32	4.74	15	46	18
EZ38_H	41	-42	68	134	4.63	-1.61	7.02	16	48	22
F252_H	40	-66	64	106	3.73	-1.54	6.49	15	47	26
F353_H	43	-59	63	133	4.23	-1.49	6.04	15	41	26
F712_H	41	-46	55	129	3.75	-1.41	5.42	17	41	18
F912_H	43	-65	68	117	4.09	-1.45	5.74	15	47	34
Lo1123_H	40	-58	63	119	4.11	-1.54	6.50	16	48	38
P465P_H	46	-59	69	138	3.99	-1.35	4.96	15	54	32
PH207_H	43	-60	64	119	3.07	-1.28	4.41	15	42	11
PHG83_H	41	-46	67	125	3.05	-1.50	6.15	17	44	13
W64A_H	38	-37	61	130	3.15	-1.29	4.44	16	45	22
